# Response to the Reply on behalf of the ‘Permanent Senate Commission for the Investigation of Health Hazards of Chemical Compounds in the Work Area’ (MAK Commission) by Andrea Hartwig Karlsruhe Institute of Technology (KIT)

**DOI:** 10.1186/s12989-015-0112-6

**Published:** 2016-01-08

**Authors:** Peter Morfeld, Joachim Bruch, Len Levy, Yufanyi Ngiewih, Ishrat Chaudhuri, Henry J. Muranko, Ross Myerson, Robert J. McCunney

**Affiliations:** 1 Institute for Occupational Epidemiology and Risk Assessment of Evonik Industries, Essen, Germany; 2 Institute and Policlinic for Occupational Medicine, Environmental Medicine and Preventive Research, University of Cologne, Cologne, Germany; 3 University Clinics Essen, Essen, Germany; 4 Cranfield University, Cranfield, UK; 5 Orion Engineered Carbons GmbH, Cologne, Germany; 6 Cabot Corporation, Billerica, MA USA; 7 Muranko & Associates, Scottsdale, AZ USA; 8 Department of Occupational Health, MedStar Washington Hospital Center, Washington, DC USA; 9 The George Washington University School of Public Health, Washington, DC USA; 10 Massachusetts Institute of Technology, Cambridge, MA USA; 11 Brigham and Women’s Hospital, Boston, MA USA

Prof. Hartwig commented [1] as chair of the MAK Commission on Morfeld et al. 2015 [2]. We would like to thank the Commission for commenting on our review. However, the MAK Commission did not address a number of important issues raised in our paper:

- Calculation error in the MAK Commission’s document on GBS^1^ [3] when using the rule of three in Pauluhn’s volumetric model (we emphasize that the comment did not dispute the arithmetical error lowering Model B’s GBS limit value erroneously from 2.0 mg/m^3^ to 0.5 mg/m^3^).
- Use of an MPPD2 program version in [3] that is outdated and no longer available to enable to replicate the MAK Commission’s conclusions.
- Input values in [3] that cannot be reproduced from the references listed in [3] or are not state-of-the-art.
- Inconsistent use of varying input data by the MAK Commission in [3] although explicitly specified as guideline in the same document [3].

In addition, the post-hoc density adaptation cannot be justified by clearance rates varying with substance density (so done in [1]) because the MAK Commission applied a constant clearance rate in all calculations [3], i.e., the rate was set invariant despite the large variation in substance densities.

We would like to draw attention again to the adverse outcome pathway (AOP) used in our paper which we feel important. Our AOP analysis revealed species-specific differences on the molecular and cellular level (see pages 20–26 in [2]).These differences are not considered in the MAK Commission’s Models A and B [3]. The unique susceptibility of rats to lung overload effects of GBS indicates that rats alone are not a good model for the derivation of GBS limit values in humans. Studies showed that other species, such as mice and hamsters, reacted differently to GBS compared to rats. Please note that relevant human data from extensive clinical studies broadly support our AOP considerations (see pages 19 and 20 in [2]). Epidemiological studies in workers exposed to various types of GBS including coal dust, carbon black and TiO_2_ did not find an excess lung cancer risk that could be related to workplace exposures (see pages 16–19 in [2]). These studies indicate that the mechanism of fibrogenic and tumorigenic effects observed in rats does not necessarily apply to humans and other species. Epidemiological evidence does not seem to have been discussed by the MAK Commission in their documentation [1] for a biological plausibility check of the derivation of the GBS limit value and GBS cancer classification.

We still feel that the particle surface area metric should have been considered by the MAK Commission as an additional third model in [3] as there is abundant published evidence that particle surface area has also been shown to significantly drive pulmonary effects of GBS.

Similarly, we think it important to note that human data give no indication of a retarded alveolar clearance under high exposure conditions as found in coal miners and feel that the Commission should have given some consideration to this well-known finding. Thus, the concept of volumetric overload of alveolar macrophages indicated by retarded alveolar clearance is not well supported by human data as the main mode of action of GBS in dust exposed workers.

Finally, although we address the errors in the calculation of the deposition fraction related to Model B in [4], the authors only validated Model A by two unnamed experts, not Model B. This validation for Model A is not relevant for our arguments. The same applies when Hartwig [1] commented on the “density division” performed in Model A by discussing the density dependence of limit values in Model B.

We would like to state that we are not criticising the use of translational models *per se* in such an important and complex area such as developing a generic approach to recommending an OEL for GBS. In fact, quite the opposite: We very much applaud the use of such models and in particular, the MAK Commission for advancing the science in this area but, at the same time, we are pleading that the output from such translational modelling is both reproducible and transparent and uses the most appropriate input data. In addition, the translational model should be used together with, rather than instead of, the most appropriate animal and relevant epidemiological finding. Such a weight of evidence approach based on concordance of findings from differing but complementary fields will best deliver robust conclusions.

In the following we address relevant issues in a point-to-point manner. We cite and number comments from [1] and respond to these passages.

COMMENT 1 in [1]:

“2. In their opinion, considerable shortcomings were:

- Dimension of lung surface area
- Lung clearance rates
- Particle deposition fractions
- Particle mass and volumetric metrics as opposed to the particle surface area metric
- Particle density”

RESPONSE 1:

One important issue about Model B is neither mentioned nor discussed. See the headline on page 9 in Morfeld et al. [2]: “*Model B: The standardization by rat lung mass or rat body weight is varying and inconsistent*”. We concluded on page 10: “*Obviously, units are confused and Morrow’s overload threshold should also refer to a 1 kg rat and, thus, should have been set to 4.2 μl per kg-rat in equation (7). This correction increases the estimated NOAEC by a factor of 4.2.*” Thus, we identified a relevant error within Model B because the “rule of three” was applied incorrectly in [4] and, consequently, by the MAK Commission [3]. The Commission [1] did not address this important issue.

COMMENT 2 in [1]:

“… was extrapolated to the human equivalent concentration calculating the NOAE particle dose deposited in the lung employing the MPPD (multi-path particle dosimetry) model Version-V2.0 program. All the input data for this calculation are given in the appendix of the MAK documentation and have been justified in detail in the MAK documentation.”

RESPONSE 2:

We were unable to obtain version 2.0 of this program, which appears to be outdated and the hyperlinks presented by the MAK Commission [3] are no longer available for others to use. Thus, a reader will be unable to reproduce the calculations because he/she is unable to get the program version V2.0 that the MAK Commission [3] applied. However, an updated version (Version 2.11) is accessible (http://www.ara.com/products/mppd.htm). We showed that Version 2.11 returns different results than Version 2.0 when using the input data listed in [3] (see e.g., Table 4 on page 12 in [2]).We stated on page 11 in [2]: “*We emphasize that a revision of the deposition calculations in* [4] *and* [3] *is needed because the deposition fractions were calculated with an MPPD version (i.e. MPPD Version 2.0) that is outdated. Hence the calculations of the MAK Commission are not based on a state of the art technique. We note that the outdated MPPD Version 2.0 is no longer publicly accessible to enable an independent reviewer to reproduce the results. Fortunately, one of the co-authors of this review has a copy of the outdated version which we used for our calculations.*”

COMMENT 3 in [1]:

“One approach (Model A) the MAK Commission used to derive a limit value … To assure that the approach is correctly described, two experts from two different institutions have been asked to independently use the model and the respective input data to calculate the deposited particle dose. Using the MPPD model and the input data described in the MAK documentation, both experts derived at the same deposited dose values as described in the MAK documentation.

Thus, the statement by Morfeld et al. [2] that they were unable to reproduce the deposited dose is not justified. Obviously, Morfeld et al. [2] used different input data and thus consequently obtained different deposited doses than the MAK Commission.”

RESPONSE 3:

We note that this is a section addressing Model A. Thus, Prof. Hartwig’s report [1] on a cross check by two experts relates only to Model A’s deposition fractions. Note further that our criticism on wrongly derived deposition fractions was not directed to Model A but to Model B. We showed that the deposition fractions used in Model B cannot be reproduced with the input data listed in the MAK document. The following headline was published on page 11 in [2]:“*Model B: Deposition fractions applied in Pauluhn* [4] *cannot be reproduced with the MPPD program given MAK’s input data* [3]”. See also Table 4 on page 12 in [2] for details on Model B deposition fractions. Thus, our criticism is on Model B. Hartwig [1] does not address this important issue about Model B at all. We demonstrated convincingly that the deposition fractions used in Model B cannot be reproduced using the input data listed in [3]. To demonstrate the problem, we draw the reader’s attention to pages 11 and 13 of [2]. We discussed the input parameter “Oronasal-Normal Augmenter” recommended by the MAK Commission: “*We emphasize that “Oronasal-Normal Augmenter” is a recommendation made in the MAK document (*[3]*, p. 58 and Appendix)… In contrast, in Pauluhn* [4] *the breathing pattern chosen was “oronasally breathing humans” (p. 186). When we, however, interpreted this as the program option “Oronasal-Mouth Breather”, MPPD 2.0 returned a deposition fraction of 16.4 % identical to that reported in* [4]*.*” Secondly, we documented that Model B calculations did not use the MAK Committee’s recommendation of an “inhalability adjustment” for rats: “*We can reproduce the value of 7.5 % published in Pauluhn* [4] *if we switch off the “inhalability adjustment” in MPPD 2.0. The MPPD tutorial explains: ‘Choose whether the program should adjust for inhalability of the aerosol using logistic functions suggested by Menache et al.* [5] *for small laboratory animals. For small particles, this inhalability is unity. By default, adjustment for inhalability is turned off.’ (MPPD 2.11 Tutorial 1: Monodisperse for Rat,* *http://www.ara.com/products/mppd.htm*). We surmise that the default option of the program was used in [4] although use of the “*inhalability correction*” *has been recommended by Oller and Oberdörster* [6] *and it is listed by the MAK Commission as the option to choose (*[3]*, p. 58).*” Thus, it is obvious that the Model B deposition fractions cannot be reproduced using the input parameters listed in MAK Commission’s document [3].

COMMENT 4 in [1]:

“The MAK Commission used the value of alveolar lung surface area of 57.22 m^2^ for humans; this is the lung volume at the end of a normal exhalation and can be determined by mechanical lung function measurements as the Functional Residual Capacity [7, 8]. The MAK Commission extensively discussed the study of Gehr et al. [9] and came to the conclusion that the value of Gehr of 143 m^2^ represents the lung area after maximal inhalation and is not realistic for workplace conditions. The MAK Commission states that the value of 57.22 m^2^ is in the lower range of the published values [3].

Gehr et al. [9] themselves give an explanation why the determined alveolar surface area in their study does not represent the “true” value: “On the other hand it is known that the epithelial surface (defined in the publication as surface membrane of alveolar epithelial cells) does not correspond to the true alveolar surface in the living air-filled lung; this surface is rather formed by the air-fluid interface of the surface lining layer which smoothes the epithelial surface. We have shown on rat lungs that the “true” alveolar surface available for gas must be by 25–50 % smaller than the epithelial surface depending on the level of air space inflation. If this is taken into consideration the “true” alveolar surface of the human lungs included in this study is reduced to 70–100 m^2^…” In their study the lungs were “fully inflated to near total lung capacity” and this inflation was achieved by instilling the fixative in aqueous solution into the airways. This does not represent the real alveolar surface area for a lightly working person and therefore this value was not taken into consideration by the MAK Commission.”

RESPONSE 4:

We would like to emphasize that the main issue under discussion about lung surface area is not the absolute area value for humans but the ratio of lung surface areas between humans and rats. This is because the lung surface area ratio is used in Model A to translate findings from experimental rats to workers. Thus, we have to consider both rat and human surface area data. Firstly, we showed in Morfeld et al. ([2], page 5) that the rat surface area chosen by the MAK Commission did neither address the rat strain under discussion (Fischer rats) nor can the value of the rat lung surface area be reproduced from the references given in [3]. We note that the MAK Commission [1] did not address this substantial issue concerning rat lung surface area data. Secondly, the surface areas should be determined with the same method to derive a reliable human/rat ratio. If the human data is normalized to “working conditions” the same must be done with the rat data. If only human data are adapted without commensurate adaption of rat data, a bias is introduced in the calculations. Note that the need of such a correction factor for rats is obvious from the passage of Gehr et al. [9] cited above in [1].Thus, the approach of the MAK Committee is biased. Thirdly, we mentioned that the data of Gehr et al. [9] are the gold standard recommended for carrying out such comparisons. Besides, data are available for the rat strain of interest (Fischer rats). Note that the MAK Commission employed data derived from an individual Long Evans rat. The overview given in Table 2 in [2] showed that an unbiased estimate of the human/rat ratio based on state-of-the-art methods is 349, not 193 as applied in [3]. Furthermore, we like to emphasize that Prof. Hartwig [1] misinterpreted the cited passage of Gehr et al. [9]. We wrote on page 6 in Morfeld et al. [2]: “*It is clear that Gehr and colleagues* [9] *discussed the surface available for gas exchange and not the epithelial surface. We note that the latter is relevant as the denominator in Model A’s metric. Furthermore, Gehr et al.* [9] *discussed the variation of the alveolar surface area in dependence on the air space inflation and Gehr’s argument relies on the assumption mentioned in* [9] *that the ratio between human and rat lung surfaces does not vary with air space inflation. Thus, the derived ratio of 349 remains valid irrespective of what degree of air space inflation is assumed to define ‘true’ values.*”

COMMENT 5 in [1]:

“The average clearance half-time value for humans of 400 days was calculated by Bailey et al. [10] and Kreyling and Scheuch [11]. The same value was used also for the derivation of ‘General Threshold of Dust’ [12].

The study of Gregoratto et al. [13] calculates a clearance half-time for humans of 300 days. ‘About 40 % of an insoluble material deposited in the alveolar-interstitial region remains sequestered indefinitely and slowly clears only to the lymph nodes. The remaining material is cleared with half-time of about 300 days.’ The model is based on studies with long-lived radionuclides uranium-238, plutonium-239, americium-241 and cobalt-60. The authors do not state clearly whether the calculation based on this model is valid for other insoluble or poorly soluble particles.”

RESPONSE 5:

The material addressed is described in the first sentence of the abstract of Gregoratto et al. [13]: “*New information on particle retention of inhaled insoluble material …*”. And in the introduction the authors said on page 353: “*The highly insoluble nature of the inhaled material …*”. Thus, it is obvious that the calculations based on these models developed by Gregoratto et al. [13] apply to poorly soluble dusts (GBS). Gregoratto et al. [13] wrote on p. 555 on best half-time estimates: “*Equivalently, the best estimates for the transfer rates from the alveolar compartment to the interstitium and to the bronchiolar region to be used in the new model are mI = 0.0010 d* ^*−1*^ *and mT = 0.0017 d* ^*−1*^ *, respectively.*” It follows that for the clearance half times: alveolar to interstitium = ln(2)/0.0010 days^−1^ = 693 days (about 700 days); alveolar to bronchial region = ln(2)/0.0017 days^−1^ = 408 days (about 400 days); from the alveolar compartment (overall) = ln(2)/0.0027 days^−1^ = 257 days (about 255 days). We conclude that the half time of 400 days used by the MAK Commission [3] and presented in Bailey et al. [10] does not consider the clearance into the interstitium (Dr. Bailey co-authored the paper by Gregoratto et al. [13]).We note that the interstitium plays no causal role for GBS effects, neither in MAK Commission’s Model A nor in Model B. This is so because MAK Commission’s Models A and B limit all adverse effects to an interaction of deposited dust with structures/cells within the alveolar compartment. Thus, in calculations based on Models A and B we have to consider the overall clearance rate from the alveolar compartment, not the clearance rate from the alveolar region to the bronchial region. This overall alveolar clearance rate is described by a half time of 255 days, not 400 days. A value of 250 days for this overall alveolar clearance half time is supported additionally by the general allometric scaling procedure proposed by West et al. [14].

COMMENT 6 in [1]:

“The mean deposition fractions were calculated for rats and humans by applying the MPPD (multi-path particle dosimetry) Version-V2.0 program. All the input data for this calculation are given in the appendix of the MAK documentation; therefore the approach of the MAK Commission is transparent and the results should be easily reproducible.”

RESPONSE 6:

Deposition fractions as used in Model B cannot be reproduced with the MAK Commission’s input data given in [3] (see RESPONSE 3). Furthermore, as already stated above (RESPONSE 2), no reader will be able to get Version 2.0 because the hyperlinks presented by the MAK Commission [3] do not lead to this version. We searched extensively and tried to receive the Version 2.0 from the internet but we failed. This version had been retracted. Only the updated Version 2.11 is available.

COMMENT 7 in [1]:

“The MAK Commission based the derivation of the MAK value on the principle that inflammation and particle retention kinetics are largely driven by the volumetric particle dose. Since phagocytosis of GBP depends primarily on their size and not on their density, the most appropriate manner to compare various dusts appears to be the volume of the material [15].

The data available to the MAK Commission did not provide values for the surface area of the particles as metric. The BET surface area is generally used as a surrogate for particle surface area. However, the issue of surface area measurement in practice is complicated. Therefore and because it is not known what part of the BET surface may be the biological/toxicological relevant particle surface, this parameter was not chosen as a dose metric by the MAK Commission. Also, according to Borm et al. [16] the debate between “surface” and “volume” metric is still ongoing.”

RESPONSE 7:

In our publication [2], we did not advocate the sole application of the particle surface area metric for the derivation of the HEC. We criticized the MAK Commission’s total disregard of the contributing effect of the retained particle surface area metric to pulmonary effects despite scientific evidence to the contrary. We stated on page 16 in [2]: “*In summary, based on our review, retained surface area appears to be a reliable unifying denominator to assess pulmonary toxicity due to exposure to GBS. The most critical question to consider in using translational toxicology with any particulate substance however, is deciding on which of the many physico-chemical properties it may possess are most relevant (see Table Three in* [17]*). Thus, the weight of evidence indicates that no one metric can be applied to all GBS substances. In particular the findings with BaSO*_*4*_ *, a GBS, challenge the basic assumptions of MAK’s translational toxicology models. … Thus, the approach of the MAK Commission which dismisses the particle surface area metric and does not test which metric is more appropriate under various circumstances appears unconvincing* [3]*.*”

COMMENT 8 in [1]:

“According to Morrow and Mermelstein [18] ‘The volumetric amount of dust available for phagocytosis is the significant factor in dust overloading: consequently a correction should be made in comparing dust of high and low density.’

In general, the retained particle dose is determined through the deposited particles minus the particles which are taken up by the macrophages and have been carried away. The highest inhaled particle mass below overload is 6 % of the macrophage volume, which is considered as NOAEC. The deposited particle mass on the lung surface takes a certain volume, depending on the density. From this particle volume the macrophages can only phagocytose a portion which will fill up 6 % of their volume. Because the particle mass of TiO_2_ with a density of 4.3 represents a different particle volume at NOAEC than the particle volume of a particle with the density of 1, the 6 % of macrophage volume will contain different masses of particles depending on their densities. Therefore, at the same mass concentration (mg/m^3^) the particles with low density give rise to higher volume than the particles with higher density. As a consequence, the particle clearance and therefore the retained particle dose is not dependent on the particle density per se but on the particle volume (Density = mass/volume).”

RESPONSE 8:

The last sentence of the above comment on density is incorrect and contradicts the logic of Model A as described in the MAK Commission’s document [3]. The MAK Commission applied alveolar clearance rates invariant of “density” and “volume” (given the same species) as we already have noted on page 5 in [2]: “*According to the findings described in Bellmann et al.* [19]*, Muhle et al.* [20] *and Pauluhn* [4] *MAK applied identical elimination half times in rats of 60 days for toner and TiO*_*2*_ *despite the different densities of both substances (see for toner equation (5) on page 55 and the calculation for TiO*_*2*_ *on page 57 in* [11]*). The implication is that, besides the particle deposition fraction, particle clearance is also independent of ‘density’.*” We note that this is true in both model applications, A and B: the half times of alveolar clearance are always set to 60 days in rats, independently of substance density. This is also made clear in Prof. Hartwig’s comment [1] on Morfeld et al. [2]. Hartwig [1] wrote above: “*For rats the clearance half-time used is 60 days* [15] *(see also figure 5 in “General Threshold of Dust”* [21]*.*” This is contradictory. A density division cannot be justified by claiming that clearance rates depend on substance density whereas the MAK Commission simultaneously used identical clearance rates for substances with very different densities.

We emphasize that our criticism of the post-hoc “density correction” is related to Model A only. Note that Model A is a retained particle mass per alveolar surface area model. It is surprising that Hartwig [1] discussed volume Model B in this section. We acknowledged that Model B leads to a density dependent limit: “*We note that in contrast to Model A, the particle density is a necessary term in this equation, and HECs derived by Model B will be density dependent*” ([2], page 8). Importantly, we have shown that the logic of Model A is incompatible with a “density adjustment” of the derived HECs. See pages 4 and 5 in [2] for a detailed demonstration of MAK’s errors and note the headline: “*Model A: Inconsistent post-hoc density adjustment*”. The MAK Commission [1] did not respond to this critical issue of Model A.

COMMENT 9 in [1]:

“Altogether, the MAK proposal of an OEL for GBP has been discussed comprehensively, taking into account all available epidemiological, experimental and mechanistic data, a scientific procedure always applied by the MAK Commission to substances under evaluation.”

RESPONSE 9:

We are unable to comment on this point as such discussions, which no doubt will have taken place, were not included in the MAK Commission’s Documentation [1]. However, to address this critical issue we presented sections on epidemiology and exposure data in [2]. We concluded on page 19: “*In summary, no causative link between exposure to well-investigated respirable GBS (including some nanostructured dusts) such as coal mine dust, TiO*_*2*_ *, toner or CB and no excess in lung cancer risk in humans has been demonstrated.*” We added on page 27: “*As reported in the Section on epidemiology above, no cancer excess risk has been found under these exposure conditions. It appears that the use of epidemiological evidence should be considered in the derivation of occupational exposure limits like those of GBS. This may also help to define the most relevant dust metric for the measurement of work environment exposures.*”

## Footnotes

1 granular biopersistent dust without known specific toxicity
